# A Comprehensive Study of Polyurethane Potting Compounds Doped with Magnesium Oxide Nanoparticles

**DOI:** 10.3390/polym15061532

**Published:** 2023-03-20

**Authors:** Jaroslav Hornak, Jakub Černohous, Pavel Prosr, Pavel Rous, Pavel Trnka, Anton Baran, Štefan Hardoň

**Affiliations:** 1 Department of Materials and Technology, Faculty of Electrical Engineering, University of West Bohemia, 306 14 Pilsen, Czech Republic; 2 Department of Physics, Faculty of Electrical Engineering and Informatics, Technical University of Košice, Park Komenského 2, 042 00 Košice, Slovakia; 3 Department of Physics, Faculty of Electrical Engineering and Information Technology, University of Žilina, 010 26 Žilina, Slovakia

**Keywords:** broadband dielectric spectroscopy, dielectric relaxation, magnesium oxide, nanocomposites, nuclear magnetic resonance, polyurethane, tensile strength

## Abstract

Recently, polyurethanes (PURs) have become a very promising group of materials with considerable utilization and innovation potential. This work presents a comprehensive analysis of the changes in material properties important for PUR applications in the electrical industry due to the incorporation of magnesium oxide (MgO) nanoparticles at different weight ratios. From the results of the investigations carried out, it is evident that the incorporation of MgO improves the volume (by up to +0.5 order of magnitude) and surface (+1 order of magnitude) resistivities, reduces the dielectric losses at higher temperatures (−62%), improves the thermal stability of the material, and slows the decomposition reaction of polyurethane at specific temperatures (+30 °C). In contrast, the incorporation of MgO results in a slight decrease in the dielectric strength (−15%) and a significant decrease in the mechanical strength (−37%).

## 1. Introduction

Polyurethanes (PURs) represent a technically significant group of polymers with a wide range of uses [1]. The basic advantage of PURs is that, at a reasonable price, a wide variety of properties and parameters are possible with simple changes in composition, while the actual polymerization reaction usually occurs at room temperature [2]. A certain disadvantage is the need to exclude moisture until the material is cured and the fact that PURs do not have the same stability at high temperatures [3] as, e.g., silicones, epoxies, or cross-linked polyester resins. Higher long-term operating temperatures (above 90 °C) acting on polyurethanes have a significant negative effect on the material properties and primary mechanical properties, and pyrolysis can occur at temperatures above 200 °C. It can therefore be argued that thermal stability is a limiting factor for the application of these materials [4,5,6]. Focusing on the positive properties mentioned above, these are mainly due to the two-phase microstructure composed of hard and soft segments [5,7]. It can thus be argued that a PUR is a block copolymer with alternating soft and hard segment components along the macromolecular chain [8]. The soft segments (polyol) contribute to the elasticity, toughness, and resiliency of the material, and the hard segments (diisocyanate and chain extender) provide physical cross-linking points, with the ratio of these segments affecting the final properties of the synthesized material [7,9,10,11].

As has already been mentioned, PURs are, in most cases, two-component systems whereby the individual components are supplied separately in liquid or semi-liquid states and the final curing process occurs only after they have been properly mixed together [12,13,14]. Common 2K systems are simply PURs or epoxies and silicones. The components are usually mixed at the point of application, immediately prior to use. The mixing produces a liquid reactive mixture in which a gradual chemical reaction occurs between the two components (isocyanate and hydroxyl groups); the reaction forms a carbamate (urethane) group (initially dissolved in the unreacted components) [15]. The viscosity of the mixture gradually increases until it gels and gradually solidifies completely. This process is slow enough to allow the low-viscosity reactive mixture to be poured into a mould with a component, e.g., an electrical component, to be poured in.

**Figure 1:**
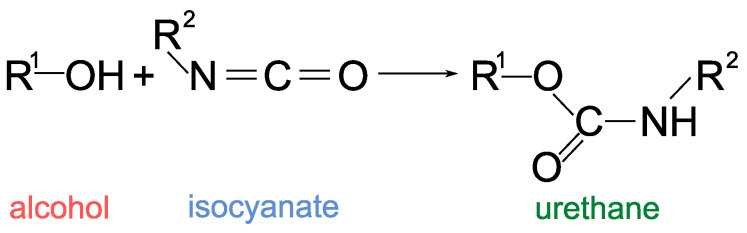
General reaction of the isocyanate groups in the hardener molecule with the hydroxyl groups of the polyol for polyurethane synthesis.

In the case of 2K PUR systems, component A is an organic polyol (a polyunsaturated alcohol, usually with various additives), and component B is an isocyanate-based hardener. Chemically, the essence of the curing is the addition reaction of the isocyanate groups in the hardener molecule with the hydroxyl groups of the polyol, according to the general equation represented in Figure 1.

The result of the above reaction equation is the already mentioned urethane group (ester of carbamic acid) [16]. In the case of multifunctional components, the product is a polymer, in this case polyurethane. If the polyol molecule contains at least three reactive OH groups and the isocyanate molecule contains at least two isocyanate groups [17,18], then the reaction (formation of urethane linkages between the OH groups and the isocyanate groups) results in a cross-linked thermoset-type polymer, i.e., a non-melting polymer without decomposition that is insoluble in solvents [19,20].

The reaction of aromatic isocyanates with aliphatic alcohols or polyols (in the sense of the above equation) proceeds slowly at room temperature. However, isocyanates (such as MDI [21]) also react with water; in addition to the solid products, CO_2_ gas is produced [22], which is undesirable (bubble formation that leads to foaming); therefore, the 2K PUR system must be protected from moisture until after curing [23].

The most basic of the additives added to the polyol component in practice is the moisture absorber (e.g., *p*-toluenesulfonyl isocyanate) [24]. Another common ingredient is a filler. The objective reasons for using fillers are primarily economic (saving organic matter); however, a suitably chosen filler mechanically strengthens the PUR, increases the thermal conductivity, suppresses the flammability of the material, or otherwise improves its properties [25,26,27,28]. A substantial increase in the hardness and strength of a PUR can be achieved by adding organic cross-linkers (substances that also react with the hardener and densify the created 3D molecular network of the polymer) [29,30].

The polyol component of modern 2K PUR systems is often a complicated mixture of substances with a carefully optimized composition in modern, sophisticated materials. Polyol also contains solid ingredients (finely powdered), which sediment during storage [31]. Before mixing the components of the 2K PUR system, it is therefore necessary to thoroughly mix and homogenize the polyol component in the container so that all possible sediment is dispersed in the volume. Only then is the hardener added (amount weighed according to the mixing ratio, which must be determined as precisely as possible), and the entire reactive mixture is thoroughly homogenized, making it ready for use.

Magnesium oxide in the form of nanoparticles was used as a modifier of polyurethane potting compounds in this research. It is an inorganic material belonging to the group of single metal oxides, with a molar mass of 40.31 g/mol [32] and a density of 3.58 g/cm^3^ [33]. It is a white hygroscopic solid mineral with empirical formula MgO, and its lattice consists of Mg^2+^ ions and O^2−^ ions linked by an ionic bond in a 1s^2^2s^2^p^6^ and 1s^2^2s^2^p^6^ configuration (d-orbitals are empty) [34]. Due to its properties (non-toxicity, environmental friendliness, superiro insulating properties), MgO is applicable in a wide range of public sectors such as industry, medicine, or biotechnology. A comprehensive description of the synthesis, characterization and selected technical applications of MgO can be found in recent studies presented by Hornak [35] or Fernandes et al. [36].

The main objective of this research is to describe and emphasize the effect of a filler (magnesium oxide nanoparticles) on the mechanical, dielectric, and structural properties of two types of polyurethanes. This is a unique comprehensive review of results that can be used especially with regard to tailoring material properties for a wide range of applications, not only in the field of high-voltage technology.

## 2. Materials

### 2.1. Polyurethane Matrices

**Figure 2:**
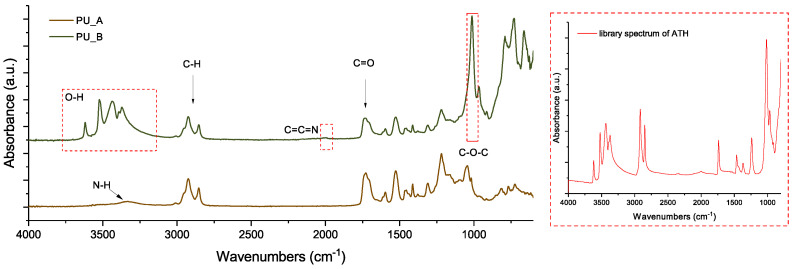
Comparison of FTIR spectra of PU_A and PU_B mixtures with the ATH library spectrum.

In this study, the properties of two polyurethane mixtures (produced by VUKI a.s., Bratislava, Slovakia) were compared (VUKOL O22, denoted PU_A, and VUKOL O33n, denoted PU_B). Both materials are based on the same polyol (castor oil) with the same drying agent additive, cured with VUKIT M (polyisocyanates based on diphenylmethane diisocyanate). VUKOL O22, however, does not contain fillers, while VUKOL O33n has a significant filler content (aluminium hydroxide—ATH [37]), which is used to obtain its fire retardant properties. This difference in the investigated materials is presented in the FTIR spectra in Figure 2, which also correspond with the spectral analyses presented in previous studies [38,39,40,41]).

The above structural differences also result in different mixing ratios of the polyol base material and hardener of 1:0.37 [42] and 1:0.23 [43], respectively. The main difference between the two materials lies in the viscosity and hardness. VUKOL O22 is a low- to medium-viscosity (max. 1500 mPa·s) grey-green polyurethane potting compound characterized by the flexibility of hard rubber (Shore A 70) and a high resistance to radiation and moisture after curing. VUKOL O33n is a high-viscosity (up to 6000 mPa·s) green-coloured polyurethane potting compound that is flame retardant, characterized by a high hardness (Shore A 87) after curing. Both compounds are solvent-free and can be safely operated in class B temperatures (130 °C).

### 2.2. Magnesium Oxide Filler

This investigation was performed with MgO nanoparticles (Nanostructured & Amorphous Materials, Inc., Garland, TX, USA) with a purity of 99%. The rest of the composition is impurities, e.g., Ca, K, or Na. The selected MgO nanoparticles have an ellipsoidal and spherical morphology and an average size of 20 nm. The specific surface area is less than 60 m^2^/g, and the bulk density is between 0.1 and 0.3 g/cm^3^. MgO powder has a white colour. Detailed characterization of these MgO particles can be found in our previous study [44].

### 2.3. PUR/MgO Preparation and Fundamental Optical Characterization

**Figure 3:**
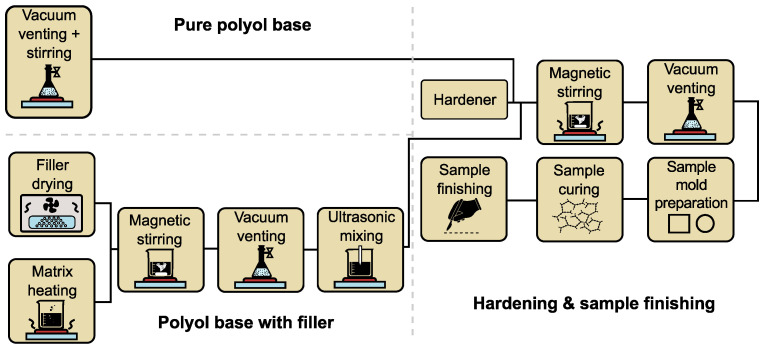
Scheme of preparation of PU_MgO nanocomposites.

**Figure 4:**
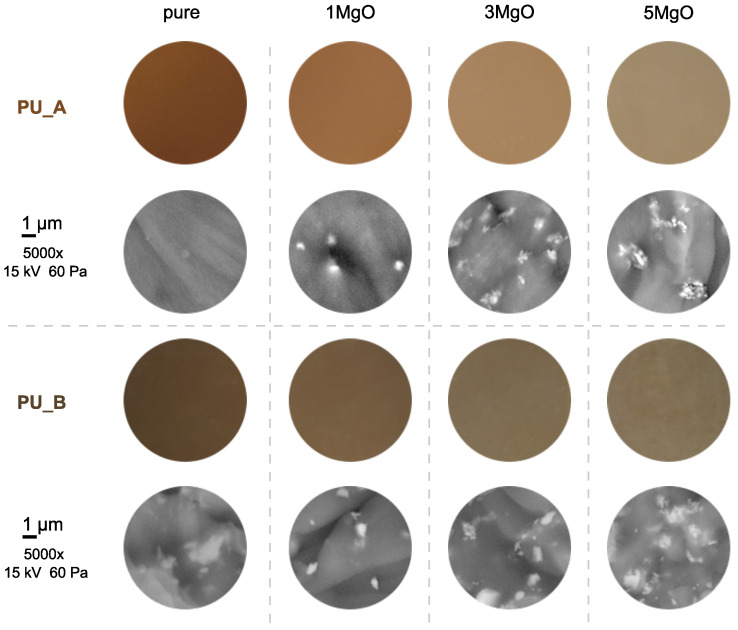
Colour change of investigated PUR composites and particle dispersion analyses of PURs with different concentrations of MgO.

The preparation of the nanocomposites was carried out under laboratory conditions, and the direct dispersion method [45] was used to produce them (Figure 3). The MgO particles, before their incorporation into the polymer base, were dried in a laboratory hot air oven for 24 h to eliminate their surface moisture. To achieve a lower viscosity for the direct blending procedure, the base component of the polyol was heated to 45 °C. To achieve an ideal concentration of nanoparticles, a precise weight of the filler MgO (0, 1, 3 and 5 wt%) was added. The mixing process of the base polyol and MgO nanoparticles was carried out for 5 h with a magnetic stirrer (700 rpm) and then combined with a vacuum process (8 mbar, 60 rpm). During this time, all air voids in the sample were removed. Once this process was completed, an ultrasonic needle was immediately applied with simultaneous magnetic stirring (60 rpm) for 60 min. This step led to further controlled dispersion [46,47]. This method was used since it guarantees dispersion of the nanoparticles in the polymer matrix, even when working with very low concentrations. Next, a hardener was added to the mixture at the recommended ratio (PU_A 1:0.37; PU_B 1:0.23). The finished mixture was vacuumed and then poured into flat square moulds with dimensions of 100 × 100 × 1.0 ± 0.2 mm. The final step was to cure these samples under laboratory conditions (24 °C, 53% R.H.) for 48 h. All sets of prepared samples contained at least four samples with the same nanoparticle concentration for repeat measurements and confirmation of the measured results. An overview of the created samples is shown in Figure 4.

A Phenom ProX Desktop scanning electron microscope (Thermo Fisher Scientific, Waltham, MA, USA) with energy-dispersive X-ray diffraction was used for simple particle dispersion analysis, as shown in Figure 4. The microscope has a backscattered electron detector (BSD), with a voltage source up to 15 kV. Two vacuum modes allow observation of uncoated samples in the lower vacuum mode. The analysis shows the presence of agglomerates on the order of a few microns, which are evenly distributed. The size and uniformity of the agglomerates vary with the filler content within the polymer matrix, which applies to both studied materials and the final shape is most likely due to the predominance of MgO particles with ellipsoidal morphology. From previous experience [26,48], an inferior level of filler dispersion is expected from the beginning of the experiment, as a simple oxide filler is purposefully used without any surface treatment. It is of course possible to apply a surface treatment for better particle dispersion [49,50,51], but the relatively high viscosity (related to the polymer molecular weight) of the base material is more important in this case [52,53]. On the other hand, the high viscosity (up to 6000 mPa·s) can also have a positive effect, especially in preventing sedimentation [54] of the filler during the curing process, which proceeds for a relatively long time at ambient temperature.

**Figure 5:**
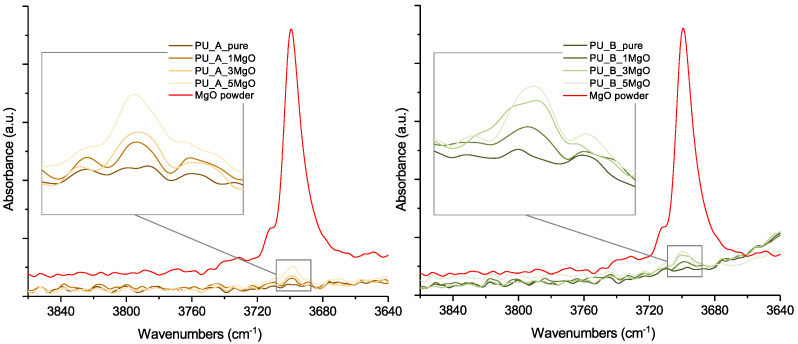
FTIR analyses of PURs with different concentrations of MgO close to the 3697 cm^−1^ spectral band region.

The presence of the MgO filler was confirmed by Fourier transform infrared spectroscopy measurements (FTIR) in the middle-IR region. Infrared spectra were measured by a Nicolet 380 spectrometer (Thermo Scientific) using a Smart MIRacle single-reflection ATR cell (diamond crystal), where 32 average scans with a resolution of 4 cm^−1^ were collected for every measured spectrum in the frequency range of 4000–600 cm^−1^. The subsequent spectral analysis was performed by OMNIC software. The presence of the MgO filler can be confirmed based on the position of the spectral band near 3697 cm^−1^, attributed to the stretching of H–O–H [55]. As shown in Figure 5, this spectral band increases with increasing MgO filler content for both tested materials. According to the Beer–Lambert law, the absorbance of IR light measured at a constant path length is directly proportional to the concentration of the sample [56]. Constant conditions were maintained during the measurements, including the contact force of the sample on the measuring crystal, to minimize significant changes in the optical path.

## 3. Methods

### 3.1. Nuclear Magnetic Resonance Spectroscopy Measurements

The broad line nuclear magnetic resonance (BL ^1^H NMR) [57,58] spectra were measured on a Varian 400 MHz solid-state NMR spectrometer (Palo Alto, CA, USA) using a probe head with 4 mm ZrO_2_ rotors at an ambient temperature of 23 °C. The BL ^1^H NMR spectra were recorded at a ^1^H resonance frequency of 400 MHz, and the chemical shifts in all spectra were referenced to tetramethylsilane using adamantane as the external standard. A π/2 pulse with a 2.9 µs duration, a 6 s recycle delay and a 20 ms acquisition time were applied during measurements.

### 3.2. Thermogravimetric Analyses

Measurement of the thermal stability of a material was performed by thermogravimetric analysis (TGA) [59] using an SDT Q650 simultaneous TGA/DSC apparatus manufactured by TA Instruments. The samples (12 mg) were placed in open platinum crucibles and exposed to a linear heating rate of 10 °C·min^−1^), starting from ambient conditions (25 °C) and increasing to 700 °C under a nitrogen atmosphere (100 mL·min^−1^). The atmosphere was changed to dry air, and the organic residue was incinerated at a temperature range between 700 and 800 °C. Thermogravimetric analysis was used for material characterization and comparison.

### 3.3. Broadband Dielectric Spectroscopy Measurement

Broadband dielectric spectroscopy (BDS) has been used to analyse the material response to alternating electric fields with variable frequency *f* (Hz), allowing the study of the behaviour of dielectric materials and the characterization of the individual contributions of polarization processes, which can be defined based on changes due to various factors [60,61]. A modular measurement system was used with an Alpha A mainframe (Novocontrol Technologies, Montabaur, Germany) and a ZGS test interface. The relative permittivity $\epsilon_{r}$ (−) and dissipation factor tan δ (−) were determined in a frequency range from 0.5 Hz to 1 MHz (for each setpoint measured from the highest frequency to the lowest), and the test voltage was set to 1 V_RMS_. The measurement was carried out in a temperature range from −50 °C to 90 °C with a temperature step of 5 °C. The samples were placed between gold-plated electrodes with a diameter of 20 mm.

### 3.4. Vector Bridge Measurements

As mentioned in the subsection above, the relative permittivity and dissipation factor are base parameters for evaluating the electrical insulation conditions. The vector bridge measurement approach performs well in characterizing the polarization responses of a dielectric from a macroscopic point of view [62]. For this investigation, 2830/2831 dielectric analysers with the appropriate 2914 test cell (all Tettex Instruments, Switzerland) were used. All measurements were performed according to the standard IEC 62631-2-1 (1000 V, 50 Hz), and the temperature range was set from 30 °C to 90 °C.

### 3.5. Resistivity Measurements

The volume resistivity $\rho_{v}$ (Ω·cm) and surface resistivity *ρ*s (Ω) are critical dielectric parameters. Applying a DC electric field to an insulating material produces a current response that can be suitably evaluated by these parameters [63]. All measurements were performed according to standards IEC 62631-3-1 and 62631-3-2, respectively, with an applied 500 V DC field at an ambient temperature of 23 ± 2 °C. The steady-state current was always recorded at the 3600th second. Measurements were carried out with a Keithley 6517A electrometer and a Keithley 8009 electrode system with an active surface area of 22.9 cm^2^ (both Keithley Instruments, Cleveland, OH, USA). All samples were conditioned in short-circuit boxes for 24 h before the measurement.

### 3.6. Dielectric Strength Measurements

The dielectric strength *Ep* (kV·mm^−1^) is one of the key parameters of insulation materials, particularly with regard to ensuring the safe and reliable operation of a device [64]. The measurements were performed according to IEC 60243-1 at a power frequency of 50 Hz with a constant increasing voltage rate of 1 kV·s^−1^. A high-voltage setup from Highvolt (Dresden, Germany) was used for the measurements. This equipment allows testing up to 220 kV AC. To eliminate surface discharge activity and surface breakdowns, the samples were immersed in mineral transformer oil. The measurements were carried out at an ambient temperature of 23 ± 2 °C.

### 3.7. Tensile Tests

The mechanical properties of the PU samples were investigated on a LabTest 3.030 universal testing machine (LaborTech, Opava, Czech Republic). The maximum stress $\sigma_{m}$ (MPa) and strain $\epsilon_{m}$ (%) at maximum were evaluated. The measurements were performed on “dog bone” samples at an ambient temperature of 23 ± 2 °C. All measurements were carried out according to ISO 527. Five samples per collection were subjected to tensile tests.

## 4. Results and Discussion

### 4.1. Effect of MgO on the Structural Properties of Polyurethane

#### 4.1.1. Nuclear Magnetic Resonance Spectroscopy

The shape of the BL ^1^H NMR spectrum of polymers in the solid state strongly depends on the strength of ^1^H–^1^H dipolar interactions between neighbouring nuclei. The molecular motion within polymers can average these interactions, which results in signal narrowing. Below the glass transition temperature (*Tg*), molecular motion in polymers is strongly suppressed, and the BL ^1^H NMR spectrum usually consists of one broad line due to the presence of strong dipolar interactions between ^1^H nuclei in rigid polymer chains. At temperatures close to and above *Tg*, the increasing intensity of molecular motion averages dipolar interactions and results in narrowing of the broad line and formation of one or more narrow lines in the BL ^1^H NMR spectrum. A broad line is assigned to hydrogen nuclei localized in rigid polymer chains, while narrow lines are associated with hydrogen nuclei in more mobile polymer chains [65].

**Figure 6:**
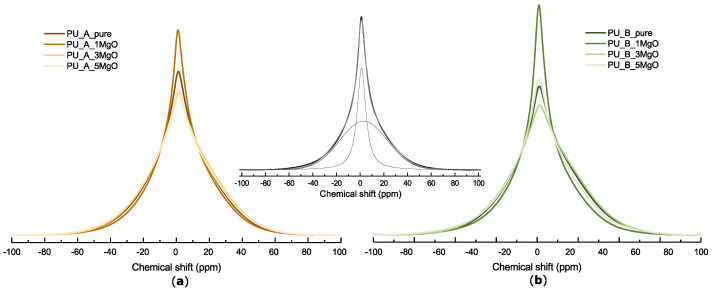
Normalized BL ^1^H NMR spectra of PU_A_pure (**a**), PU_B_pure (**b**) and their nanocomposites with 1, 3 and 5 wt.% MgO nanoparticles measured at ambient temperature. The inset in the figure shows deconvolution of the normalized BL ^1^H NMR spectrum of PU_B_1MgO into broad and narrow lines.

Figure 6 depicts the BL ^1^H NMR spectra for PU_A, PU_B and their nanocomposites measured at ambient temperature. All spectra are superimpositions of one broad and one narrow line, which are, respectively, related to hydrogen nuclei in the rigid structure—hard (diisocyanate) segments— and hydrogen nuclei in mobile polymer chains—soft (polyol) segments. The widths of the broad and narrow lines for each spectrum estimated from the deconvolution of BL ^1^H NMR spectra are listed in Table 1. The width of the lines was determined with an error of 2%. The distinct increase in the narrow line intensity together with the decrease in the widths of both the narrow and broad lines (Table 1) observed in the BL ^1^H NMR spectra for both composites (PU_A and PU_B) at the lowest concentration of MgO nanoparticles reveals enhanced polymer chain mobility in these nanocomposites. The increase in polymer chain mobility due to mixing of MgO into the polymer matrix can be caused by both an increase in phase separation and weaker soft–hard chain interactions. The hydrogen bonds between the NH groups of urethane and MgO nanoparticles are responsible for the interaction between nanoparticles and polymer chains in hard domains. This interaction and the proper distribution of nanoparticles inside the polymer increase the phase separation of hard and soft domains, which can lead to an increase in the free volume in the polymer and an increase in polymer chain motion. The interaction between the NH groups of urethane and MgO nanoparticles decreases the number of available NH groups required for the formation of hydrogen bonds with the ethereal groups of soft segments; hence, the soft segment mobility increases due to the decrease in hydrogen bonds with hard segments [66]. On the other hand, both spectra of the PU_A composites with 3 and 5% MgO nanoparticles are wider than the spectrum of PU_A_pure, and an obvious decrease in the narrow line intensities is also observed for both composites. This reveals the opposite effect on the mobility of polymer chains of higher MgO nanoparticle contents. Similar behaviour was also observed for PU_B composites with 3% MgO nanoparticles, except for samples with 5% MgO, in which mixing of MgO particles only slightly influenced the shape of the BL ^1^H NMR spectrum. At higher concentrations, MgO nanoparticles presumably form agglomerates that introduce restrictions on polymer chain motion, resulting in an increase in the line widths in NMR spectra for samples with higher nanofiller content.

#### 4.1.2. Thermogravimetric Analyses

**Figure 7:**
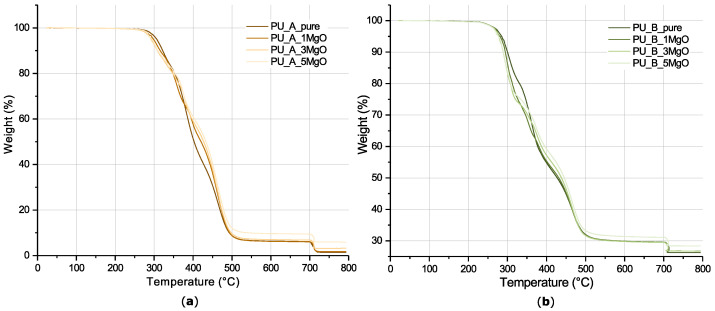
Comparison of TGA curves for tested samples: (**a**) sample set PU_A; (**b**) sample set PU_B.

From the literature, it is obvious that the thermal decomposition of polyurethanes occurs in several steps [4]. As mentioned in [67], the main decomposition reaction of pure PU occurs at 200–400 °C, followed by decomposition of substituted urea at 400–800 °C. Based on the thermogravimetric (TGA) curves (Figure 7), it can be concluded that PU_A has a higher thermal stability compared to PU_B. The onset temperature of the first decomposition reaction corresponds to 291 °C for PU_A, while for PU_B, it corresponds to 278 °C. Another difference between the tested materials is the different residual mass after complete thermal decomposition. For PU_A almost complete thermal decomposition has taken place, whereas for PU_B the residual mass corresponds to approximately 26%. This is due to the confirmed presence of ATH in PU_B, whose thermal decomposition does not result in complete loss [68]. Comparing the TGA curves of the modified samples, there is a visible shift of the weight loss of the doped composites at approximately 400 °C to a higher temperature. The cause of this shift may be due to the retardation properties of the MgO filler, whose thermal decomposition, according to [69,70], occurs in the temperature range of approximately 400 °C. A higher content of MgO results in increasing thermal stability in this temperature range and slowing of the decomposition reaction. The opposite behaviour with increasing amount of filler is seen in the case of the PU_B material. Based on FTIR analysis, the presence of ATH was detected in this set of samples. According to [71], the derivative of the weight loss of ATH reaches its maximum at 262 °C. A higher proportion of this component is therefore reflected by a decrease in the onset decomposition temperature. The trend in the region around 400 °C is identical to that of the previous material.

### 4.2. Effect of MgO on the Dielectric Properties of Polyurethane

#### 4.2.1. Volume Resistivity and Surface Resistivity

**Figure 8:**
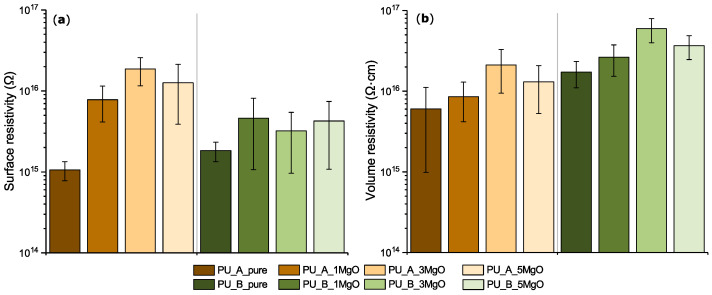
Interaction of PU_A and PU_B composites with a DC field: (**a**) surface resistivity; (**b**) volume resistivity.

The volume and surface resistivities are very important parameters in terms of insulation properties. From the results in Figure 8, it can be seen that when magnesium oxide is incorporated into the polyurethane matrix, both the internal and surface resistivities increase. This positive finding is confirmed by the fact that the nanoparticles, or their agglomerates, are uniformly dispersed in the material [72]. Studies [73,74] have highlighted the positive effect of nanometric size fillers on the distribution of the trapped charge in the internal structure of the material, especially at low loading levels.

This can be attributed to the fact that some carriers are trapped in deep traps (these traps affect the charge transfer in the interface region [75]) and space charge is gradually accumulated in the electrode region, resulting in limited charge carrier injection [76,77,78]. This situation then results in a dynamic equilibrium of the system reflected in a stable electric field, resulting in a lower conductivity of the whole system. Due to the nature of the particles, whose bulk resistivity is on the order of 10^19^ (Ω·cm) [79], the resistivity of the whole composite can increase at low filling levels. In addition, it can also be assumed that nanoparticles may also act against internal foaming formation, cavity formation, or free volume decrease [80,81], while these changes in the structure are also evident from the BL ^1^H NMR results. A decrease in volume and surface resistivity is expected at higher filling levels [82,83]. In the presented case, a decrease in volume resistivity occurs when the 3% level is reached. It is most likely due to exceeding of percolation threshold [84] of the composite. This means that the level of filler concentration has exceeded the level where the material begins to lose its electrical insulating ability due to the excessive amount of additives, associated with the formation of a conductive pathway along their surface [85,86].

#### 4.2.2. Relative Permittivity and Dissipation Factor under Different Measurement Conditions

**Figure 9:**
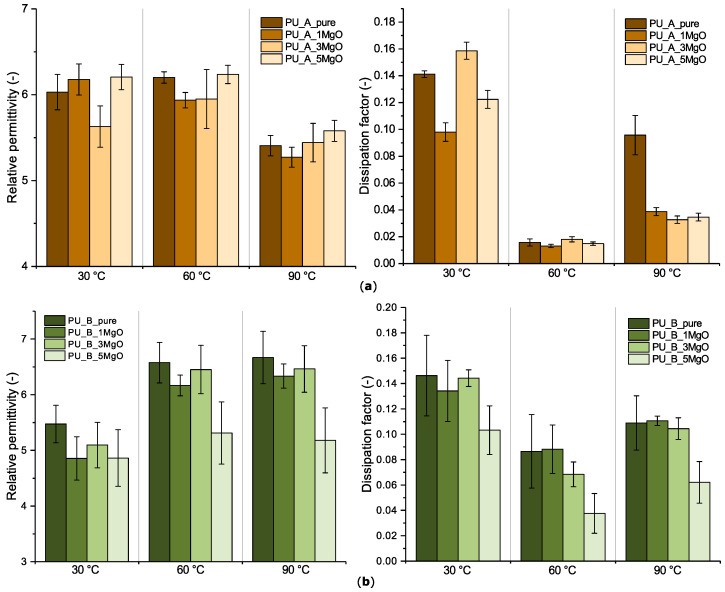
Dielectric properties of polyurethane mixtures with MgO nanoparticles measured via VB: (**a**) sample set PU_A; (**b**) sample set PU_B.

The results of the dissipation factor and relative permittivity obtained by vector bridge measurement-VB-(1000 V, 50 Hz) are presented in Figure 9. From these results, it can be seen that there is a noticeable difference between the studied mixtures, not so much in terms of absolute values as in terms of the temperature dependence of each parameter.

As seen from these graphs, the relative permittivity of PU_B increased, whereas that of PU_A showed a slight decrease at 90 °C (valid for all sample compositions). This decrease is mainly due to strong thermal motions that disturb the orientation of the individual dipoles in the material structure [87]. A significant effect of the filling level on the relative permittivity is not visible for all studied materials. The most significant change is for material PU_B_5MgO, but the differences are within the range of the population standard deviations and are highlighted in particular by the scale of the individual bar graphs. If attention is given to the dissipation factor, then there is a noticeable change in the temperature region around 60 °C, with a sharp decrease for PU_A and a slight decrease for PU_B. This behaviour has been observed in previous studies [26,48,88]. Most likely, this can be attributed to the easier breaking of the intermolecular forces between the polymer chains, and the polar groups can move more easily due to the effect of the higher temperature [87].

**Figure 10:**
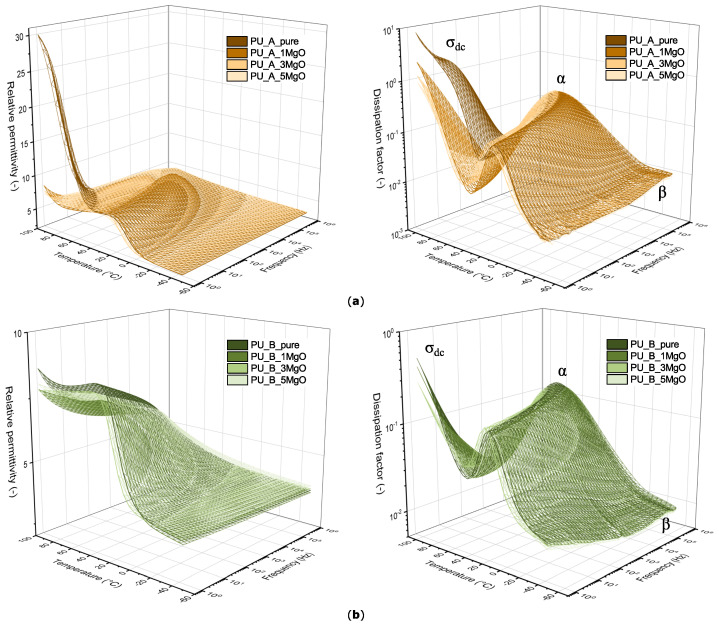
Dielectric properties of polyurethane mixtures with MgO nanoparticles measured via BDS: (**a**) sample set PU_A; (**b**) sample set PU_B.

A more comprehensive view of the behaviour of the studied polyurethanes in the temperature–frequency region is provided by the results from broadband dielectric spectroscopy-BDS-(1 V, 0.5 Hz to 1 MHz). From the surface plots presented in Figure 10, it can be seen that a significant conductivity $\sigma_{dc}$ component of the PU_A_pure mixture appears in the low-frequency and high-temperature region.

A positive finding is that the addition of MgO nanoparticles most likely shifts this conductivity component into the higher-temperature region. This conductivity component, to which Maxwell–Wagner–Sillars (MWS) polarization contributes to some extent, most likely overlays the onset of the peak of hard-segment relaxation ($\alpha_{H}$ relaxation) [88]. Focusing on the other features that are apparent from the results, there is a broad peak from approximately −20 °C to 40 °C for PU_A mixtures and from 0 °C to 60 °C for PU_B mixtures. A similar behaviour was observed in the study by Boiteux et al. [88], whereby this relaxation (α relaxation) is caused most likely by a mixed phase of hard and soft block segments of polyurethane. In the region of lower temperatures and higher frequencies, the contribution of β relaxation is almost negligible. This relaxation is associated with the local motion of chain segments. It is most likely attributed to the motion of the polar carbonyl groups that form the structural base of polyurethane [89]. A relatively similar behaviour of polyurethane in a temperature–frequency field, but at different temperature ranges, has been described in other previously published studies [90,91,92,93,94], and this behaviour is influenced by many factors, such as the type and amount of polyol, water content, chain extenders, or chain end groups. The slight differences in the absolute values obtained via BDS and VB, which are negligible, could be due to differences in the voltage level, sample diameter, or sensitivity of the measuring device.

#### 4.2.3. Breakdown Voltage Measurement

**Figure 11:**
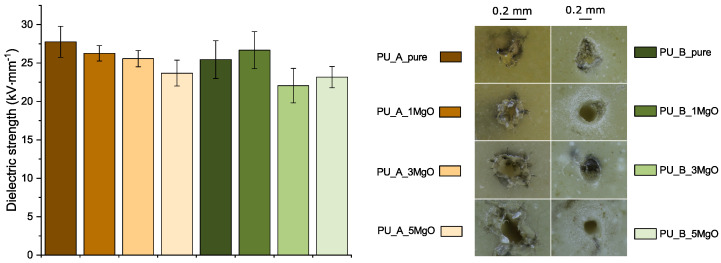
Dielectric strength results and demonstration of breakdown channels.

It is evident from the measurement results (Figure 11) that there is no significant positive effect of MgO addition with respect to improvement of the dielectric strength. The microscopic images show the difference between PU_A and PU_B. The PU_B mixture has a more uniform shape of the breakdown channel with a larger diameter. This is most likely due to the higher hardness of the base material. The addition of nanoparticles to both mixtures has no other observable effect according to the optical inspection performed.

The differences in dielectric strength are on the order of standard deviations of the individual datasets. In contrast, a slight decrease in the electrical strength of the PU_A mixtures with increasing MgO concentration can be observed. This trend is not confirmed for PU_B, but the measurements show a larger variation in these values, which is acceptable. A similar behaviour was presented, for example, in the study by Ersoy et al. [95]. These changes in the dielectric strength can probably be attributed to the higher amount of moisture in the material structure due to the MgO addition [96]. According to this study, the slight increase in electrical strength for PU_B_1MgO may be because the mean free path of water molecules for passing through the network may increase due to the incorporation of MgO. In other situations, the dominant phenomenon is probably the moisture absorption of MgO. In this case, an assumption of three layers around the particle is made. The first layer contains approximately 5–10 H_2_O molecules strongly bound to the surface of the particle (one molecule of H_2_O measures 0.278 nm in size) [97]. The second layer of H_2_O may be weakly bound by van der Waals bonds. The third layer is formed by water, which is present in the polymer base. In particular, the first and second layers could contribute to the formation of conductive channels [98] for charge carriers, resulting in easier electrical breakdown.

### 4.3. Effect of MgO on the Mechanical Properties of Polyurethane

**Figure 12:**
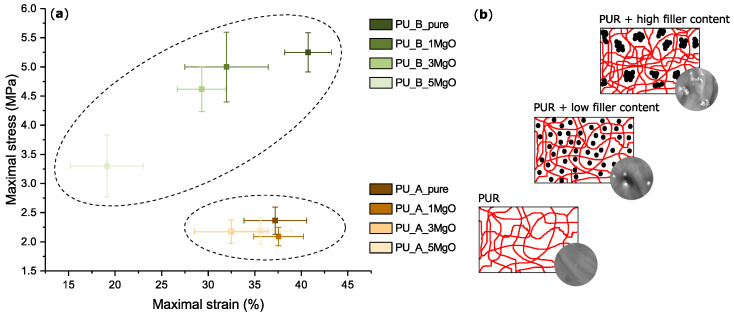
Mechanical properties of polyurethane mixtures with MgO nanoparticles: (**a**) tensile test results; (**b**) interactions of PURs with MgO (inspired and redrawn from: [99]).

It can be seen from the results presented in Figure 12 that the mechanical properties of the two mixtures are fundamentally different but correspond to the dataset values [42,43]. It can also be seen from this graph that as the MgO filling level increases, there is a gradual decrease in the maximum stress $\sigma_{m}$ (MPa) and strain $\epsilon_{m}$ (%) at maximum, especially for the PU_B mixture. For the second mixture PU_A, there is also a deterioration in the mechanical properties, but it is not very pronounced (changes are within the range of standard deviations).

These decreases in the mechanical properties can be attributed to the aforementioned agglomerates of MgO nanoparticles effectively restricting the mobility of polymer chains in polyurethane [99]. In a study [100], D’Orazio et al. explained this behaviour as follows: the molecular motions of the hard segments of polyurethane are limited by the presence of the inorganic phase of MgO. The assumption of molecular scale contiguity between polyurethane hard segments and MgO results in a reduction in the mobility of the chain segments and increases the cooperative nature of the corresponding relaxation process. This subsequently leads to a reduction in the number of interactions between the hard and soft segments in the polyurethane matrix and changes in the mechanical properties, which have already been confirmed by other studies [8,101,102].

## 5. Conclusions

Two different commercially available polyurethanes for electrical applications were modified by magnesium oxide addition at different weight percentages. From the findings, the following can be concluded: (i) magnesium oxide improves the electrical insulating properties, especially with respect to the volume and surface resistivities of the PUR; (ii) magnesium oxide shifts the conductivity component $\sigma_{dc}$ of the PUR to the higher-temperature region, which was confirmed by BDS measurements, and this phenomenon was also confirmed by vector bridge measurements of the dissipation factor; (iii) magnesium oxide has no positive impact on the dielectric strength of PUR/MgO composites; (iv) magnesium oxide deteriorates the mechanical properties of the PUR with increasing filling level, with respect to both the tensile strength and relative elongation of the material; (v) magnesium oxide increases the thermal stability and slows the decomposition reaction of the PUR.

## Figures and Tables

**Table 1:** Widths of broad (BL) and narrow (NL) lines of the BL ^1^H NMR spectra corresponding to hard and soft segments, respectively, measured for PU_A_pure, PU_B_pure and their nanocomposites. Intensities of particular lines are shown in parentheses.

Line	PU_A				PU_B			
Width	Pure	1MgO	3MgO	5MgO	Pure	1MgO	3MgO	5MgO
BL (kHz)	20.2	18.7	20.3	21.2	21.8	18.9	22.1	21.4
	(0.73)	(0.70)	(0.84)	(0.78)	(0.77)	(0.64)	(0.80)	(0.73)
NL (kHz)	5.0	3.7	4.6	4.9	5.0	3.5	6.1	5.0
	(0.27)	(0.30)	(0.16)	(0.22)	(0.23)	(0.36)	(0.20)	(0.27)

## Data Availability

The raw/processed data required to reproduce these findings cannot be shared at this time due to technical or time limitations.

## References

[1] Szycher M. (2013). Polyurethanes. Szycher’S Handbook of Polyurethanes.

[2] Volkova E.R., Tereshatov V.V., Karmanov V.I., Makarova M.A., Slobodinyuk A.I. (2013). Polyurethane adhesive composition cured at room temperature. Polym. Sci. Ser. D.

[3] Liu S.H., Shen M.Y., Kuan C.F., Kuan H.C., Ke C.Y., Chiang C.L. (2019). Improving Thermal Stability of Polyurethane through the Addition of Hyperbranched Polysiloxane. Polymers.

[4] Pagacz J., Hebda E., Janowski B., Sternik D., Jancia M., Pielichowski K. (2018). Thermal decomposition studies on polyurethane elastomers reinforced with polyhedral silsesquioxanes by evolved gas analysis. Polym. Degrad. Stab..

[5] Amado J.C.Q., Evingür G.A., Pekcan Ö., Achilias D.S. (2019). Thermal Resistance Properties of Polyurethanes and Its Composites. Thermosoftening Plastics.

[6] Gaboriaud F., Vantelon J.P. (1982). Mechanism of thermal degradation of polyurethane based on MDI and propoxylated trimethylol propane. J. Polym. Sci. Polym. Chem. Ed..

[7] Kumagai S., Motokucho S., Yabuki R., Anzai A., Kameda T., Watanabe A., Nakatani H., Yoshioka T. (2017). Effects of hard- and soft-segment composition on pyrolysis characteristics of MDI, BD, and PTMG-based polyurethane. JAAP.

[8] Bugrov A.N., Gorshkova Y.E., Ivan’kova E.M., Kopitsa G.P., Pavlova A.A., Popova E.N., Smirnova V.E., Smyslov R.Y., Svetlichnyi V.M., Vaganov G.V. (2022). Domain Structure, Thermal and Mechanical Properties of Polycaprolactone-Based Multiblock Polyurethane-Ureas under Control of Hard and Soft Segment Lengths. Polymers.

[9] Jiang L., Ren Z., Zhao W., Liu W., Liu H., Zhu C. (2018). Synthesis and structure/properties characterizations of four polyurethane model hard segments. R. Soc. Open Sci..

[10] Jin X., Guo N., You Z., Tan Y. (2020). Design and Performance of Polyurethane Elastomers Composed with Different Soft Segments. Materials.

[11] Klinedinst D.B., Yilgör I., Yilgör E., Zhang M., Wilkes G.L. (2012). The effect of varying soft and hard segment length on the structure–property relationships of segmented polyurethanes based on a linear symmetric diisocyanate, 1,4-butanediol and PTMO soft segments. Polymer.

[12] Liu H., Bi Z., Wan Z., Wang X., Wan Y., Guo X., Cai Z. (2020). Preparation and Performance Optimization of Two-Component Waterborne Polyurethane Locomotive Coating. Coatings.

[13] Vaca M.L.A., Gonzalez J.S., Hoppe C.E. (2022). Soft Elastomers Based on the Epoxy–Amine Chemistry and Their Use for the Design of Adsorbent Amphiphilic Magnetic Nanocomposites. Macromol.

[14] Saeedi I.A., Andritsch T., Vaughan A.S. (2019). On the Dielectric Behavior of Amine znd Anhydride Cured Epoxy Resins Modified Using Multi-Terminal Epoxy Functional Network Modifier. Polymers.

[15] Petrović Z.S., Ferguson J. (1991). Polyurethane elastomers. Prog. Polym. Sci..

[16] Ouellette R.J., Rawn J.D. (2015). Synthetic Polymers. Principles of Organic Chemistry.

[17] Heath D.E., Guelcher S.A., Cooper S.L. (2013). Polyurethanes. Biomaterials Science: An Introduction to Materials in Medicine.

[18] Janik H., Sienkiewicz M., Kucinska-Lipka J. (2014). Polyurethanes. Handbook of Thermoset Plastics.

[19] Ma S., Webster D.C. (2018). Degradable thermosets based on labile bonds or linkages: A review. Prog. Polym. Sci..

[20] Montarnal D., Capelot M., Tournilhac F., Leibler L. (2011). Silica-Like Malleable Materials from Permanent Organic Networks. Science.

[21] Frisch K.C. (2002). Chapter 16—Chemistry and technology of polyurethane adhesives. Adhes. Sci. Eng..

[22] Rao R.R., Mondy L.A., Long K.N., Celina M.C., Wyatt N., Roberts C.C., Soehnel M.M., Brunini V.E. (2017). The kinetics of polyurethane structural foam formation: Foaming and polymerization. AIChE J..

[23] Abram E.R., Bowler N. Effect of relative humidity on the curing and dielectric properties of polyurethane-based composites. Proceedings of the CEIDP’05. 2005 Annual Report Conference on Electrical Insulation and Dielectric Phenomena.

[24] Chen X., Hu C., Xu H., Qu R., Hu X., Yang J., Song X. (2022). Synthesized polyurethane from p-toluenesulfonyl isocyanate and epichlorohydrin via salen catalysis. Polym. Adv. Technol..

[25] Członka S., Kairytė A., Miedzińska K., Strąkowska A., Adamus-Włodarczyk A. (2021). Mechanically Strong Polyurethane Composites Reinforced with Montmorillonite-Modified Sage Filler (*Salvia officinalis* L.). Int. J. Mol. Sci..

[26] Kúdelčík J., Hardoň Š., Trnka P., Michal O., Hornak J. (2021). Dielectric Responses of Polyurethane/Zinc Oxide Blends for Dry-Type Cast Cold-Curing Resin Transformers. Polymers.

[27] Wondu E., Lule Z., Kim J. (2019). Thermal Conductivity and Mechanical Properties of Thermoplastic Polyurethane-/Silane-Modified Al_2_O_3_ Composite Fabricated via Melt Compounding. Polymers.

[28] Altafim R.A.C., Murakami C.R., Neto S.C., Araújo L.C.R., Chierice G.O. (2003). The Effects of Fillers on Polyurethane Resin-based Electrical Insulators. Mater. Res..

[29] Vedage G.A., Burdeniuc J.J., Arnold A.R., Tobias J.D. (2013). Crosslinkers for Improving Stability of Polyurethane Foams. U.S. Patent.

[30] Tramontano J., Blank W.J. Crosslinking of Water-Borne Polyurethane Dispersions. Proceedings of the 21st Annual Waterborne, High-Solids, and Powder Coatings Symposium.

[31] Ionescu M. (2016). Polymer Polyols (Filled Polyols) Chemistry and Technology of Polyols for Polyurethanes.

[32] Cheremisinoff N.P.V., Cheremisinoff N.P. (2001). Condensed Encyclopedia of Polymer Engineering Terms.

[33] Akinwekomi A.D., Tang C.-Y., Tsui G.C.-P., Law W.-C., Chen L., Yang X.-S., Hamdi M. (2018). Synthesis and Characterisation of Floatable Magnesium Alloy Syntactic Foams with Hybridised Cell Morphology. Mater. Des..

[34] Singh J.P., Chae K.H. (2017). D^∘^ Ferromagnetism of Magnesium Oxide. Condens. Matter.

[35] Hornak J. (2021). Synthesis, Properties, and Selected Technical Applications of Magnesium Oxide Nanoparticles: A Review. Int. J. Mol. Sci..

[36] Fernandes M., RB Singh K., Sarkar T., Singh P., Pratap Singh R. (2020). Recent Applications of Magnesium Oxide (MgO) Nanoparticles in Various Domains. Adv. Mater. Lett..

[37] Fan S., Zhang X., Lu Y., Gao Y. Characterization of HTV Silicone Rubber with Different Content of ATH Filler by Mechanical Measurements, FTIR and XPS Analyzes. Proceedings of the 2018 12th International Conference on the Properties and Applications of Dielectric Materials (ICPADM).

[38] Asefnejad A., Khorasani T.M., Behnamghader, Farsadzadeh B. (2011). Bonakdar Manufacturing of biodegradable polyurethane scaffolds based on polycaprolactone using a phase separation method: Physical properties and in vitro assay. Int. J. Nanomed..

[39] Diasa R.C.M., Serakides A.M.G.R., Ayresa E., Oréfice R.L. (2010). Porous Biodegradable Polyurethane Nanocomposites: Preparation, Characterization, and Biocompatibility Tests. Mater. Res..

[40] Bandekar J., Klima S. (1991). FT-IR spectroscopic studies of polyurethanes Part I. Bonding between urethane COC groups and the NH Groups. J. Mol. Struct..

[41] Dalpech M., Miranda G. (2012). Waterborne polyurethanes: Influence of chain extender in ftir spectra profiles. Open Eng..

[42] (2018). VUKI, a.s. Zalévací hmoty VUKUR. VUKOL O22. https://www.vuki.sk/files/technicke_listy/TDS-VUKOL-O22-ver-2018-04-30-sk.pdf.

[43] (2019). VUKI, a.s. Zalévací hmoty VUKUR. VUKOL O33n. https://www.vuki.sk/files/technicke_listy/TDS-VUKOL-O33-n-n-ver-2019-03-28-sk.pdf.

[44] Hornak J., Trnka P., Kadlec P., Michal O., Mentlík V., Šutta P., Csányi G.M., Tamus Z.Á. (2018). Magnesium Oxide Nanoparticles: Dielectric Properties, Surface Functionalization and Improvement of Epoxy-Based Composites Insulating Properties. Nanomaterials.

[45] Rahman M.M. (2020). Polyurethane/Zinc Oxide (PU/ZnO) Composite—Synthesis, Protective Property and Application. Polymers.

[46] Bittmann B., Haupert F., Schlarb A.K. (2009). Ultrasonic dispersion of inorganic nanoparticles in epoxy resin. Ultrason. Sonochem..

[47] Goyat M.S., Ray S., Ghosh P.K. (2011). Innovative application of ultrasonic mixing to produce homogeneously mixed nanoparticulate-epoxy composite of improved physical properties. Compos. Part A Appl. Sci. Manuf..

[48] Kúdelčík J., Hardoň Š., Hockicko P., Kúdelčíková M., Hornak J., Prosr P., Trnka P. (2020). Study of the Complex Permittivity of a Polyurethane Matrix Modified by Nanoparticles. IEEE Access.

[49] Palimi M.J., Rostami M., Mahdavian M., Ramezanzadeh B. (2014). Surface modification of Fe_2_O_3_ nanoparticles with 3-aminopropyltrimethoxysilane (APTMS): An attempt to investigate surface treatment on surface chemistry and mechanical properties of polyurethane/Fe_2_O_3_ nanocomposites. Appl. Surf. Sci..

[50] Guo Z., Park S., Wei S., Pereira T., Moldovan M., Karki A.B., Young D.P., Hahn H.T. (2007). Flexible high-loading particle-reinforced polyurethane magnetic nanocomposite fabrication through particle-surface-initiated polymerization. Nanotechnology.

[51] Sabzi M., Mirabedini S.M., Zohuriaan-Mehr J., Atai M. (2009). Surface modification of TiO_2_ nano-particles with silane coupling agent and investigation of its effect on the properties of polyurethane composite coating. Prog. Org. Coat..

[52] Choi J.Y., Park C.H., Lee J. (2008). Effect of Polymer Molecular Weight on Nanocomminution of Poorly Soluble Drug. Drug Deliv..

[53] Corbierre M.K., Cameron N.S., Sutton M., Laaziri K., Lennox B.R. (2005). Gold Nanoparticle/Polymer Nanocomposites: Dispersion of Nanoparticles as a Function of Capping Agent Molecular Weight and Grafting Density. Langmuir.

[54] Hornak J., Kadlec P., Polanský R. (2020). Halloysite Nanotubes as an Additive to Ensure Enhanced Characteristics of Cold-Curing Epoxy Resins under Fire Conditions. Polymers.

[55] Zahir M.H., Rahman M.M., Irshad K., Rahman M.M. (2019). Shape-Stabilized Phase Change Materials for Solar Energy Storage: MgO and Mg(OH)_2_ Mixed with Polyethylene Glycol. Nanomaterials.

[56] Salzer R. (2008). Peter R. Griffiths, James A. de Haseth: Fourier Transform Infrared Spectrometry.

[57] Baran A., Vrábel P., Kovaľaková M., Hutníková M., Fričová O., Olčák D. (2020). Effects of sorbitol and formamide plasticizers on molecular motion in corn starch studied using NMR and DMTA. J. Appl. Polym. Sci..

[58] Mujbil H.H., Al Jebur L.A., Yousif E., Kadhom M., Mohammed A., Ahmed D.S., Ali M., Hashim H. (2022). Utilization of Metal Oxides Nanoparticles in Modulating Polyvinyl Chloride Films to Resist Ultraviolet Light. Metals.

[59] Polanský R., Prosr P., Čermák M. (2014). Determination of the thermal endurance of PCB FR4 epoxy laminates via thermal analyses. Polym. Degrad. Stab..

[60] Havran P., Cimbala R., Király J., Rajňák M., Bucko S., Kurimský J., Dolník B. (2022). Frequency-Dependent Dielectric Spectroscopy of Insulating Nanofluids Based on GTL Oil during Accelerated Thermal Aging. Processes.

[61] Kremer F., Schönhals A. (2003). Broadband Dielectric Spectroscopy.

[62] Kao K.C. (2004). Dielectric Phenomena in Solids with Emphasis on Physical Concepts of Electronic Processes.

[63] Lee H.G., Kim J.G. (2020). Volume and Surface Resistivity Measurement of Insulating Materials Using Guard-Ring Terminal Electrodes. Energies.

[64] Kadota Y. (2022). Dielectric Breakdown from a Reliability and Safety Viewpoint. Test Navi Rep..

[65] Spěváček J., Brus J., Divers T., Grohens Y. (2007). Solid-state NMR study of biodegradable starch/polycaprolactone blends. Eur. Polym. J..

[66] Azari M., Sadeghi M., Aroon M., Matsuura T. (2019). Polyurethane Mixed Matrix Membranes for Gas Separation: A Systematic Study on Effect of SiO_2_/TiO_2_ Nanoparticles. J. Membr. Sci. Res..

[67] Shi X., Jiang S., Zhu J., Li G., Peng X. (2018). Establishment of a highly efficient flame-retardant system for rigid polyurethane foams based on bi-phase flame-retardant actions. RSC Adv..

[68] Jianjun M., Junxiao Y., Yawen H., Ke C. (2012). Aluminum–organophosphorus hybrid nanorods for simultaneously enhancing the flame retardancy and mechanical properties of epoxy resin. J. Mater. Chem..

[69] Rani N., Chahal S., Kumar P., Shukla R., Singh S.K. (2019). A comparative study on magnesium hydroxide and magnesium oxide nanostructures. Dae Solid State Phys. Symp..

[70] Akram M.W., Fakhar-e-Alam M., Atif M., Butt A.R., Asghar A., Jamil Y., Alimgeer K.S., Wang Z.M. (2018). In vitro evaluation of the toxic effects of MgO nanostructure in Hela cell line. Sci. Rep..

[71] Bi W., Sun J., Yu G., Goegelein C.H., Hoch M., Klaassen J., Kirchhoff J., Zhao S. Study on Interaction between Aluminum Hydroxide and Vinyltriethoxy Silane by Gas Chromatography-Mass Spectrometry. Proceedings of the IOP Conference Series: Earth and Environmental Science.

[72] Wenhu Y., Ran Y., Xu Y., Man X., Sisi H., Xiaolong C. (2012). Effect of Particle Size and Dispersion on Dielectric Properties in ZnO/Epoxy Resin Composites. Trans. Electr. Electron. Mater..

[73] Andritsch T., Kochetov R., Morshuis P.H.F., Smit J.J. Dielectric properties and space charge behavior of MgO-epoxy nanocomposites. Proceedings of the 2010 10th IEEE International Conference on Solid Dielectrics.

[74] Khan M.Z., Wang F., Li J., Hassan M.A.S., Ahmad J., He L., Kaizhen W. AC Breakdown Strength and Volume Resistivity Characteristics of Epoxy Resin Composite with Surface Modified Alumina Nanoparticles. Proceedings of the 2018 IEEE International Conference on High Voltage Engineering and Application (ICHVE).

[75] Wang Y., Xiao K., Wang C., Yang L., Wang F. (2016). Effect of Nanoparticle Surface Modification and Filling Concentration on Space Charge Characteristics in TiO_2_/XLPE Nanocomposites. J. Nanomater..

[76] Kyokane J., Tsujimoto N., Ishida M., Fukuma M. Space charge characteristics of fullerenol and carbon nanotube doped polyurethane elastomer (PUE) actuators. Proceedings of the 2005 International Symposium on Electrical Insulating Materials.

[77] Watanabe M., Hirai T. (2004). Space charge distribution in bending-electrostrictive polyurethane films doped with salts. J. Appl. Polym. Sci..

[78] Watanabe M., Wakimoto N., Shirai H., Hirai T. (2003). Bending electrostriction and space-charge distribution in polyurethane films. J. Appl. Phys..

[79] Andritch T. (2010). Epoxy Based Nanodielectrics for High Voltage DC-Applications—Synthesis, Dielectric Properties and Space Charge Dynamics. Ph.D. Thesis.

[80] Awad S., Al-Rashdi A., Abdel-Hady E.E., Van Horn J.D. (2018). Free volume properties of the zinc oxide nanoparticles/waterborne polyurethane coating system studied by a slow positron beam. J. Compos. Mater..

[81] Park S.H., Hwang J., Park G.S., Ha J.H., Zhang M., Kim D., Yun D.J., Lee S., Lee S.H. (2019). Modeling the electrical resistivity of polymer composites with segregated structures. Nat. Commun..

[82] Ge G., Tang Y., Li Y., Huang L. (2020). Effect of Environmental Temperature on the Insulating Performance of Epoxy/MgO Nanocomposites. Appl. Sci..

[83] Hu S., Zhou Y., Yuan C., Wang W., Hu J., Li Q., He J. (2020). Surface-modification effect of MgO nanoparticles on the electrical properties of polypropylene nanocomposite. High Volt..

[84] Shah Z.M., Khanday F.A., Malik G.F.A., Jhat Z.A. (2022). Fabrication of Polymer Nanocomposite-Based Fractional-Order Capacitor: A Guide. Fractional-Order Design.

[85] Habeeb M., Hamza R.S.A. (2018). Synthesis of (polymer blend –MgO) nanocomposites and studying electrical properties for piezoelectric application. Indones. J. Electr. Eng. Inform..

[86] Bertasius P., Meisak D., Macutkevic J., Kuzhir P., Selskis A., Volnyanko E., Banys J. (2019). Fine Tuning of Electrical Transport and Dielectric Properties of Epoxy/Carbon Nanotubes Composites via Magnesium Oxide Additives. Polymers.

[87] Ahmad Z. (2012). Polymer Dielectric Materials. Dielectr. Mater..

[88] Boiteux G., Seytre G., Cuve L., Pascault J.P. (1991). Dielectric studies of segmented polyurethanes based on polyolefine: Relations between structure and dielectric behaviour. J. Non. Cryst. Solids.

[89] Kanapitsas A., Pissis P., Gomez Ribelles J.L., Monleon Pradas M., Privalko E.G., Privalko V.P. (1999). Molecular mobility and hydration properties of segmented polyurethanes with varying structure of soft- and hard-chain segments. J. Appl. Polym. Sci..

[90] Karabanova L.V., Boiteux G., Gain O., Seytre G., Sergeeva L.M., Lutsyk E.D. (2001). Semiinterpenetrating polymer networks based on polyurethane and polyvinylpyrrolidone. I. Thermodynamic state and dynamic mechanical analysis. J. Appl. Polym. Sci..

[91] Pissis P., Apekis L., Christodoulides C., Niaounakis M., Kyritsis A., Nedbal J. (1996). Water effects in polyurethane block copolymers. J. Polym. Sci..

[92] Oprea S., Potolinca O., Oprea V. (2010). Dielectric properties of castor oil cross-linked polyurethane. High Perform. Polym..

[93] Madbouly S.A., Kessler M.R. Dielectric spectroscopy for biorenewable plant oil-based polyurethane. Proceedings of the 2014 IEEE Conference on Electrical Insulation and Dielectric Phenomena (CEIDP).

[94] Pissis P., Kanapitsas A., Savelyev Y.V., Akhranovich E.R., Privalko E.G., Privalko V.P. (1998). Influence of chain extenders and chain end groups on properties of segmented polyurethanes. II. Dielectric study. Polymer.

[95] Ersoy A., Hiziroglu H.R. Electrical breakdown of polyurethane-based nanocomposites. Proceedings of the 2010 10th IEEE International Conference on Solid Dielectrics.

[96] Ha Thuc C.N., Cao H.T., Nguyen D.M., Tran M.A., Duclaux L., Grillet A.-C., Ha Thuc H. (2014). Preparation and Characterization of Polyurethane Nanocomposites Using Vietnamese Montmorillonite Modified by Polyol Surfactants. J. Nanomater..

[97] Butyrskaya E., Nechaeva L., Shaposhnikov V., Selemenev V. (2015). Determining role of hydrogen bonding in electrically driven membrane transport: Quantum-chemical and molecular dynamics study. Pet. Chem..

[98] Zou C., Fothergill J.C., Rowe S.W. (2008). The effect of water absorption on the dielectric properties of epoxy nanocomposite. IEEE Trans. Dielectr. Electr. Insul..

[99] Anancharoenwong E., Chueangchayaphan W., Rakkapao N., Marthosa S., Chaisrikhwun B. (2021). Thermo-mechanical and antimicrobial properties of natural rubber-based polyurethane nanocomposites for biomedical applications. Polym. Bull..

[100] D’Orazio L., Grippo A. (2015). A water dispersed Titanium dioxide/poly(carbonate urethane) nanocomposite for protecting cultural heritage: Preparation and properties. Prog. Org. Coat..

[101] Ginzburg V.V., Bicerano J., Christenson C.P., Schrock A.K., Patashinski A.Z. (2009). Modeling Mechanical Properties of Segmented Polyurethanes. Nano- and Micromechanics of Polymer Blends and Composites.

[102] Wongsamut C., Suwanpreedee R., Manuspiya H. (2020). Thermoplastic polyurethane-based polycarbonate diol hot melt adhesives: The effect of hard-soft segment ratio on adhesion properties. Int. J. Adhes. Adhes..

